# Morphological and functional echocardiographic findings in pediatric patients diagnosed with hypermobile Ehlers-Danlos syndrome

**DOI:** 10.3389/fped.2026.1768520

**Published:** 2026-04-22

**Authors:** Walter Vignaroli, Gioia Mastromoro, Carolina Putotto, Filippo Camerota, Claudia Celletti, Mauro Celli, Luca Celli, Anna Zambrano, Francesco Carlomagno, Emanuele Monda, Annapaola Cirillo, Bruno Marino, Paolo Versacci

**Affiliations:** 1 Department of Cardiac Surgery, San Carlo di Nancy Hospital, GVM Care & Research, Rome, Italy; 2 Department of Maternal Infantile and Urological Sciences, Sapienza University of Rome, Rome, Italy; 3 Department of Experimental Medicine, Sapienza University of Rome, Rome, Italy; 4 Department of Laboratory Science, Research and Development Division, Ospedale Isola Tiberina-Gemelli Isola, Rome, Italy; 5 Physical Medicine and Rehabilitation, Sapienza University of Rome, Rome, Italy; 6 Department of Life Sciences, Health, and Health Profession, Link Campus University, Rome, Italy; 7 Inherited and Rare Cardiovascular Diseases, Department of Translational Medical Sciences, University of Campania “Luigi Vanvitelli”, Monaldi Hospital, Naples, Italy

**Keywords:** aortic dilatation, cardiovascular anomalies, echocardiography, Ehlers-Danlos syndrome, global longitudinal strain

## Abstract

**Introduction:**

Hypermobile Ehlers-Danlos syndrome (hEDS) is a connective tissue disorder that may involve the cardiovascular system. While mitral valve prolapse (MVP) and aortic root dilatation (ARD) have been documented in adults with hEDS, their prevalence in children remains unclear.

**Methods:**

This study evaluated 28 pediatric patients with hEDS and 28 age- and body surface area–matched healthy controls using two-dimensional echocardiography, Doppler, Tissue Doppler Imaging (TDI), and Speckle Tracking Echocardiography (STE), including global longitudinal strain (GLS) analysis.

**Results:**

Nine hEDS patients (32%) exhibited MVP. Although aortic root and ascending aorta Z-scores remained within the normal range, they were significantly higher in hEDS patients compared to controls. TDI revealed reduced late diastolic septal myocardial velocities (*p* = 0.004). While left ventricular ejection fraction (EF) was preserved, hEDS patients showed significantly reduced GLS values (*p* < 0.001), indicating subclinical myocardial dysfunction. hEDS diagnosis correlated independently with reduced GLS (*p* = 0.01), but not with EF.

**Discussion:**

These findings suggest that cardiac alterations such as MVP can manifest in childhood and that subtle myocardial impairment can occur despite normal EF. STE and GLS analyses are valuable tools for the early detection of functional abnormalities. Routine cardiac follow-up in pediatric hEDS patients may help identify and monitor these early cardiovascular changes, potentially improving long-term outcomes.

## Introduction

Ehlers–Danlos Syndrome (EDS) is a heterogeneous group of connective tissue disorders characterized by structural and functional alterations of the skin, ligaments, joints, and blood vessels (1). In individuals presenting with clinical features suggestive of EDS, only a limited number of pathogenic variants in genes associated with fibrillar structure can be identified. The disorder is most commonly caused by impaired synthesis of collagen types I, III, and V (2, 3), resulting in a wide spectrum of phenotypic manifestations. Collagen type I, in particular, is the most abundant collagen in human connective tissues, accounting for approximately 75% of the total collagen content in the myocardium. It plays a key role in maintaining aortic wall strength and stiffness.

The 2017 International Classification for the Ehlers–Danlos Syndromes recognizes 13 EDS subtypes (4). This document defines the diagnosis of hEDS as clinical because the molecular basis of this disorder remains unknown. Consequently, there is no definitive test available to confirm or exclude the diagnosis. During a genetic evaluation, the physician evaluates the signs and symptoms and looks for characteristics that could suggest other EDS subtypes or other collagen diseases. Specifically, two additional criteria must be met in addition to generalized joint hypermobility for a diagnosis of hEDS to be made (4).

Genetic tests do not represent a diagnostic criterion, since the molecular basis remains unknown and only a small number of hEDS cases have been attributed to haploinsufficiency of genes related to proteins expressed in connective tissues, such as *TNXB* (MIM * 600985) (4), *COL3A1* (MIM * 120180) (5), and *LZTS1* (MIM * 606551) (6).

The most common clinical features of hEDS include generalized joint hypermobility and skin hyperlaxity (7). Less frequently, patients may present with musculoskeletal manifestations, early-onset fatigue, easy bruising, and hematomas, in addition to ocular, gastrointestinal, orthopedic, and/or cardiovascular abnormalities (8). Some individuals may also experience chronic pain, dysautonomia, and anxiety (9).

Cardiovascular involvement in adult patients with hEDS has been described in several studies, most commonly including mitral valve prolapse (MVP) and mild aortic root dilatation (ARD), with an overall reported prevalence ranging approximately from 6% to 21%, depending on the studied population and diagnostic criteria (10–12). However, the prevalence and characteristics of structural and functional cardiovascular abnormalities in pediatric patients remain largely unknown (13–15).

The aim of this study was to comprehensively evaluate both morphological and functional cardiovascular abnormalities in pediatric patients with hEDS. While cardiovascular involvement—including MVP, ARD, and subtle ventricular dysfunction—is well-described in adults with hEDS, its early presence and characteristics in children are poorly defined. To address this gap, we employed conventional echocardiography alongside advanced techniques, such as Tissue Doppler Imaging (TDI) and Two-dimensional Speckle Tracking Echocardiography (STE). STE, in particular, is a sensitive, non-invasive method that allows for a detailed assessment of segmental myocardial function and the detection of subclinical abnormalities, even when left ventricular ejection fraction (EF) is normal. Identifying these early alterations is clinically relevant because they may reflect initial changes in myocardial structure and function that precede overt cardiac disease, highlighting the importance of early cardiovascular assessment in the pediatric hEDS population (16).

## Materials and methods

### Study population

From February 2021 to September 2023, 28 pediatric patients diagnosed with hEDS were evaluated in a retrospective single-center study at the Pediatric Cardiology Unit of the Department of Maternal Infantile, and Urological Sciences of Sapienza University of Rome. The diagnosis of hEDS was established by two clinicians in accordance with the 2017 International Classification for the Ehlers–Danlos Syndromes. All participants presented with generalized joint hypermobility with a score of at least 6 out of 9, unusually velvety skin, skin hyperextensibility, piezogenic papules on the heels, and chronic referred pain. None had a positive family history. Alternative diagnoses were excluded after taking the patient's medical history and performing physical examinations.

The inclusion criteria were: (1) age <18 years at the time of evaluation; (2) a diagnosis of hypermobile Ehlers–Danlos syndrome (hEDS) established according to the 2017 International Classification of the Ehlers–Danlos Syndromes; (3) availability of a complete clinical evaluation and a transthoracic echocardiographic examination performed at our institution during the study period; and (4) availability of complete medical records suitable for retrospective analysis. The exclusion criteria were: (1) a diagnosis of other subtypes of Ehlers–Danlos syndrome or other hereditary connective tissue disorders (e.g., Marfan syndrome or Loeys–Dietz syndrome); (2) the presence of previously diagnosed congenital heart disease unrelated to hEDS; (3) incomplete clinical or echocardiographic data; and (4) patients with previous cardiac surgery or interventional cardiac procedures.

The study cohort included 16 girls (57%) and 12 boys (43%), with a mean age of 11 ± 6 years (range 2–18 years).

A control group (C) of 28 subjects (8 girls, 20 boys), with no history of cardiovascular disease, was matched by age and body surface area (BSA) and enrolled for comparison (Table 1). Both patients and controls underwent transthoracic echocardiography, and myocardial function parameters, including strain values assessed by STE, were collected. Written informed consent was obtained from all participants or their legal guardians. The study complied with the Declaration of Helsinki and was approved by the local Institutional Review Boards for Human Studies (Protocol Ref. 6082).

**Table 1:** Demographic and anthropometric parameters of the study population.

	hEDS (*n* = 28)	Controls (*n* = 28)	*p*-value
Age (years)	11 ± 6	10 ± 3	0.53
Height (m)	1.4 ± 0.2	1.4 ± 0.2	1
Weight (kg)	41.7 ± 16.6	40 ± 17.8	0.75
BSA (m^2^)	1.28 ± 0.06	1.23 ± 0.06	0.66

Data are expressed as the mean ± standard deviation. *p*-values <0.05 are in bold. BSA, body surface area.

### Echocardiographic evaluations

Echocardiographic examinations were performed using an M5S-D probe (bandwidth 1.5–4.5 MHz) connected to a GE Vivid 9 echocardiography system (Medical Systems, Oslo, Norway). Measurements of the left ventricular (LV) internal diastolic diameter, interventricular septal diastolic thickness, LV posterior wall diastolic thickness, aortic root diameter, and left atrial dimensions were obtained in the parasternal long-axis view, in accordance with the recommendations of the American Society of Echocardiography (17, 18).

Proximal aortic diameters were assessed using two-dimensional imaging at four levels—namely, the aortic annulus, the sinuses of Valsalva (SVs), the sinotubular junction, and the proximal ascending aorta (AA)—during systole, in accordance with current guidelines (17, 18). All measurements were indexed to BSA, which was calculated using the Haycock formula (19). LV mass was calculated using the Devereux equation (20) and subsequently indexed to BSA and to height raised to the power of 2.7 to derive the LV mass index (21).

LV global systolic function was evaluated by calculating the ejection fraction (EF) and fractional shortening (FS) using M-mode imaging, with EF further confirmed using the Simpson biplane method. Diastolic function was assessed through mitral inflow Doppler and pulsed-wave Tissue Doppler Imaging (TDI). Mitral inflow velocities were obtained by placing the pulsed-wave Doppler sample volume at the tips of the mitral valve leaflets. Early (E) and late (A) diastolic velocities, deceleration time (DT), and the E/A ratio were measured.

TDI was performed in the apical four-chamber view by positioning the sample volume at the lateral and septal margins of the mitral annulus and at the lateral margin of the tricuspid annulus. Peak systolic (S), early diastolic (E'), and late diastolic (A') velocities were recorded for both ventricles. LV isovolumic relaxation time (IVRT) and isovolumic contraction time (IVCT) were measured at the lateral border of the mitral annulus, from the end of the S wave to the onset of the E' wave, and from the end of the A' wave to the onset of the S wave, respectively. The myocardial performance index (MPI) was calculated using time intervals derived from TDI at the lateral mitral annulus and the LV ejection time (ET), which was obtained from the aortic Doppler trace, according to the formula: MPI = (IVCT + IVRT)/ET.

Right ventricular (RV) systolic function was assessed by measuring tricuspid annular plane systolic excursion (TAPSE).

To quantify LV myocardial function, STE analysis was performed. Second-harmonic, two-dimensional images were acquired from the apical three-chamber (A3C), four-chamber (A4C), and two-chamber (A2C) views, and digital loops were stored for offline analysis using dedicated software (EchoPac; GE). The endocardial borders of the LV were manually traced at the end-systolic frame in each apical view to assess longitudinal strain (LS). The automatically generated region of interest was adjusted manually when necessary. An automated segmental analysis was then performed to derive peak strain and time-to-peak strain values.

All echocardiographic parameters were calculated as the average of three consecutive cardiac cycles (22).

### Statistical analysis

Statistical analyses were conducted using MedCalc Statistical Software version 15.8 (MedCalc Software bvba, Ostend, Belgium; https://www.medcalc.org; 2015). Data distribution was assessed using the Shapiro–Wilk test. Variables with a normal distribution (*p* > 0.05) were considered parametric, whereas those not normally distributed (*p* ≤ 0.05) were considered non-parametric.

Continuous variables with a normal distribution are expressed as mean ± standard deviation (SD), while non-normally distributed variables are reported as median and interquartile range (IQR). Categorical variables are presented as absolute numbers and percentages [*n* (%)] and are reported consecutively throughout the manuscript for clarity and consistency.

Comparisons between groups were performed using the Student's *t*-test for normally distributed continuous variables and the Mann–Whitney *U*-test for non-normally distributed variables. The Chi-square test or Fisher's exact test was used for categorical variables, as appropriate.

Correlations between variables assessing ventricular function were evaluated using the Pearson correlation coefficient for parametric variables and the Spearman's rank correlation coefficient (rho) for non-parametric variables.

To explore the independent associations between baseline characteristics and measures of ventricular function, two multivariate regression models (using a stepwise approach) were constructed, using global (G) peak LV longitudinal strain average and LV ejection fraction (Simpson's method) as dependent variables. Only variables showing significant associations in the univariate analysis were entered into the multivariate models. The goodness-of-fit of the models was assessed using the adjusted coefficient of determination (adjusted R²). A two-tailed *p*-value < 0.05 was considered statistically significant.

## Results

No significant differences were observed between the two groups with regard to demographic and anthropometric characteristics. The mean age was 11 ± 6 years in the hEDS group and 10 ± 3 years in controls (*p* = 0.53). The mean height was identical in both groups (1.4 ± 0.2 m vs. 1.4 ± 0.2 m; *p* = 1.00), as was the mean body weight (41.7 ± 16.6 kg vs. 40.0 ± 17.8 kg; *p* = 0.75). Similarly, body surface area (BSA) did not differ significantly between patients with hEDS and controls (1.28 ± 0.06 m² vs. 1.23 ± 0.06 m²; *p* = 0.66).

The demographic and anthropometric characteristics of the study cohort population are listed in Table 1.

In total, 9 of the 28 hEDS subjects (9/28, 32%) were diagnosed with MVP. Mild mitral valve regurgitation was detected in four subjects (4/28, 14%). Patent foramen ovale was observed in 2/28 subjects (7%). Ascending aortic dilatation, of mild degree in all cases, was identified in 7/28 patients (25%); one of these patients also presented with mild aortic root dilatation indexed to BSA. Z-scores of the sinuses of Valsalva (*p* = 0.016) and the ascending aorta (*p* = 0.0016) were significantly higher in the hEDS group, although mean values remained within normal limits.

Conventional echocardiography showed no significant differences in left ventricular (LV) wall thickness between groups. Global systolic function was within physiological ranges in both groups. However, a statistically significant reduction in LV systolic performance was observed in the hEDS group compared with controls, as assessed by EF (Teichholz: 63 ± 0.9, 65.6 ± 0.77, *p* = 0.036; Simpson: 62.5 ± 1.2, 65.1 ± 0.81, *p* = 0.029) and fractional shortening (33.86 ± 0.7, 35.8 ± 0.5, *p* = 0.041). Significant differences were also observed in LV end-diastolic internal diameter (40.3 ± 1, 43.4 ± 0.8, *p* = 0.018), end-diastolic volume (72.9 ± 4, 86 ± 4, *p* = 0.019), stroke volume (45.8 ± 2.4, 56.7 ± 2.7, *p* = 0.004), LV mass index (65.1 ± 4.5, 83.6 ± 3.3, *p* = 0.0017), and LVMI indexed to height^2.7 (31 ± 1.6, 37.9 ± 1.3, *p* = 0.0015). (Table 2). As expected, parameters derived from the Teichholz method are mathematically interdependent and therefore partially account for the observed differences between groups.

**Table 2:** Conventional echocardiography parameters (cardiac dimensional parameters, extracardiac structure measurements, and systolic function parameters).

	hEDS (*n* = 28)	Controls (*n* = 28)	*p*-value
LVIDd (mm)	40.3 ± 1	43.4 ± 0.8	**0**.**018**
LVIDs (mm)	26.5 ± 0.78	27.9 ± 0.6	0.16
IVSTd (mm)	7.5 ± 0.3	7.7 ± 0.2	0.67
IVSTs (mm)	10.46 ± 0.35	11.04 ± 0.37	0.27
LVPWTd (mm)	6.4 ± 0.35	6.3 ± 0.25	0.93
LVPWTs (mm)	10.75 ± 0.4	11.21 ± 0.4	0.42
EDV (ml)	72.9 ± 4	86 ± 4	**0**.**019**
ESV (ml)	27.3 ± 1.8	30 ± 1.6	0.28
EF (%)—Teichholz	63 ± 0.9	65.6 ± 0.77	**0**.**036**
FS (%)	33.86 ± 0.7	35.8 ± 0.5	**0**.**041**
SV (ml)	45.8 ± 2.4	56.7 ± 2.7	**0**.**004**
LVs mass (g)	86.05 ± 7.9	100.5 ± 8.9	0.23
LVd mass (g)	90.3 ± 8.7	106.8 ± 9.2	0.2
LV mass/BSA (g/m^2^)	65.1 ± 4.5	83.6 ± 3.3	**0**.**0017**
LVMI (g/m^2.7^)	31 ± 1.6	37.9 ± 1.3	**0**.**0015**
EF (%)—Simpson	62.5 ± 1.2	65.1 ± 0.81	**0**.**029**
Aortic annulus (mm)	16.45 ± 0.53	15.9 ± 0.4	0.5
Z-score Annulus	−0.17 ± 0.2	−0.54 ± 0.18	0.19
Sinuses of Valsalva (mm)	23 ± 0.8	21.9 ± 0.6	0.19
Z-score Sinuses of Valsalva	0.15 ± 0.17	−0.45 ± 0.17	**0**.**016**
Sinotubular junction (mm)	17.7 ± 0.7	17 ± 0.65	0.46
Z-score S-t Junction	−0.26 ± 0.18	−0.63 ± 0.17	0.15
Proximal Ascending Aorta (mm)	20.8 ± 0.8	19 ± 0.5	0.06
Z-score Ascending Aorta	1.32 ± 0.18	0.5 ± 0.17	**0**.**0016**
TAPSE (cm)	2.05 ± 0.06	2.17 ± 0.05	0.15

Data are expressed as the mean ± standard deviation. *p*-values <0.05 are in bold. LVIDd, left ventricular internal diameter at end-diastole; LVIDs, left ventricular internal diameter at end-systole; IVSTd, interventricular septum thickness at end-diastole; IVSTs, interventricular septum thickness at end-systole; LVPWd, left ventricular posterior wall thickness at end-diastole; LVPWs, left ventricular posterior wall thickness at end-systole; EDV, end-diastolic volume; ESV, end-systolic volume; EF, ejection fraction; FS, fractional shortening; SV, stroke volume; LV, left ventricular; LVMI, left ventricular mass index; TAPSE, tricuspid annular plane systolic excursion.

Tissue Doppler Imaging revealed a significant reduction in late diastolic septal mitral velocity (A' septal) in the hEDS group compared with controls (0.07 ± 0.002, 0.06 ± 0.0018, *p* = 0.004). Ejection time (ET) was also significantly reduced (291.7 ± 4.3, 318.1 ± 6.5, *p* = 0.0015).

Other parameters, including IVRT, IVCT, and right ventricular systolic function assessed by TAPSE, were comparable between groups (Table 3).

**Table 3:** Doppler and tissue Doppler imaging parameters.

	hEDS (*n* = 28)	Control (*n* = 28)	*p*-value
MV E-wave (m/s)	1.02 ± 0.03	1.03 ± 0.02	0.7
MV A-wave (m/s)	0.52 ± 0.026	0.5 ± 0.02	0.35
MV DT (ms)	154.4 ± 5.6	165.5 ± 4.8	0.13
MV E/A	2.03 ± 0.09	2.2 ± 0.1	0.2
Mitral S’ lateral (mm/s)	0.11 ± 0.05	0.22 ± 0.06	0.09
Mitral E’ lateral (mm/s)	0.2 ± 0.004	0.2 ± 0.006	0.2
Mitral A’ lateral (mm/s)	0.07 ± 0.003	0.06 ± 0.003	0.32
Mitral S’ septal (mm/s)	0.08 ± 0.003	0.08 ± 0.0018	0.5
Mitral E’ septal (mm/s)	0.15 ± 0.006	0.16 ± 0.003	0.06
Mitral A’ septal (mm/s)	0.07 ± 0.002	0.06 ± 0.0018	**0**.**004**
Tricuspid S’ lateral (mm/s)	0.13 ± 0.003	0.13 ± 0.005	0.7
Tricuspid E’ lateral (mm/s)	0.16 ± 0.005	0.17 ± 0.004	0.15
Tricuspid A’ lateral (mm/s)	0.1 ± 0.005	0.09 ± 0.005	0.0503
IVCT (ms)	49.4 ± 2.4	45.3 ± 1.8	0.17
IVRT (ms)	50.6 ± 2.6	51.9 ± 2.1	0.7
ET (ms)	291.7 ± 4.3	318.1 ± 6.5	**0**.**0015**
MPI	0.34 ± 0.1	0.33 ± 0.1	0.35

Data are expressed as the mean ± standard deviation. *p*-values <0.05 are in bold. E, E-wave mitral inflow; A, A-wave mitral inflow; E/A ratio; DT, deceleration time of the E-wave; Mitral S’ lateral, systolic tissue Doppler velocity at the lateral mitral annulus; Mitral E’ lateral, early diastolic peak velocity at the lateral mitral annulus; Mitral A’ lateral, late diastolic peak velocity at the lateral mitral annulus; Mitral S’ septal systolic tissue Doppler velocity at the septal mitral annulus; Mitral E’ septal, early diastolic peak velocity at the septal mitral annulus; Mitral A’ septal, late diastolic peak velocity at the septal mitral annulus; Tricuspid S’ lateral, systolic tissue Doppler velocity at the lateral tricuspid annulus; Tricuspid E’ lateral, early diastolic peak velocity at the lateral tricuspid annulus; Mitral A’ lateral, late diastolic peak velocity at the lateral tricuspid annulus; IVCT, isovolumic contraction time; IVRT, isovolumic relaxation time; ET, ejection time of the LV; MPI, myocardial performance index.

Longitudinal Strain (LS) analysis revealed significant differences in GLS values of the LV between the hEDS group and healthy controls for each of the examined echocardiographic views: G peak LS (A3C) (−18.5 ± 0.64, −22.3 ± 0.51, *p* < 0.0001), G peak LS (A4C) (−19.5 ± 0.6, −21.5 ± 0.62, *p* = 0.0228), G peak LS (A2C) (−20.5 ± 0.66, −23.2 ± 0.54, *p* = 0.0024), and G peak LS Average (Avg) (−19.4 ± 0.5, −22.4 ± 0.5, *p* = 0.0001) (Table 4). Despite the average GLS of all subjects being within normal limits, 7 hEDS subjects out of 28 (25%) had pathological mean GLS values (22).

**Table 4:** Peak systolic longitudinal strain analysis of global parameters.

	hEDS (*n* = 28)	Control (*n* = 28)	*p*-value
G peak LS (Average) (%)	−19.4 ± 0.5	−22.4 ± 0.5	**0**.**0001**
G peak LS (A3C) (%)	−18.5 ± 0.64	−22.3 ± 0.51	**<0**.**0001**
G peak LS (A4C) (%)	−19.5 ± 0.6	−21.5 ± 0.62	**0**.**0228**
G peak LS (A2C) (%)	−20.5 ± 0.66	−23.2 ± 0.54	**0**.**0024**

Data are expressed as the mean ± standard deviation. *p*-values <0.05 are in bold. A3C, apical 3-chamber view; A4C, apical 4-chamber view; A2C, apical 2-chamber view; G, global; LS, longitudinal strain.

**Figure 1:**
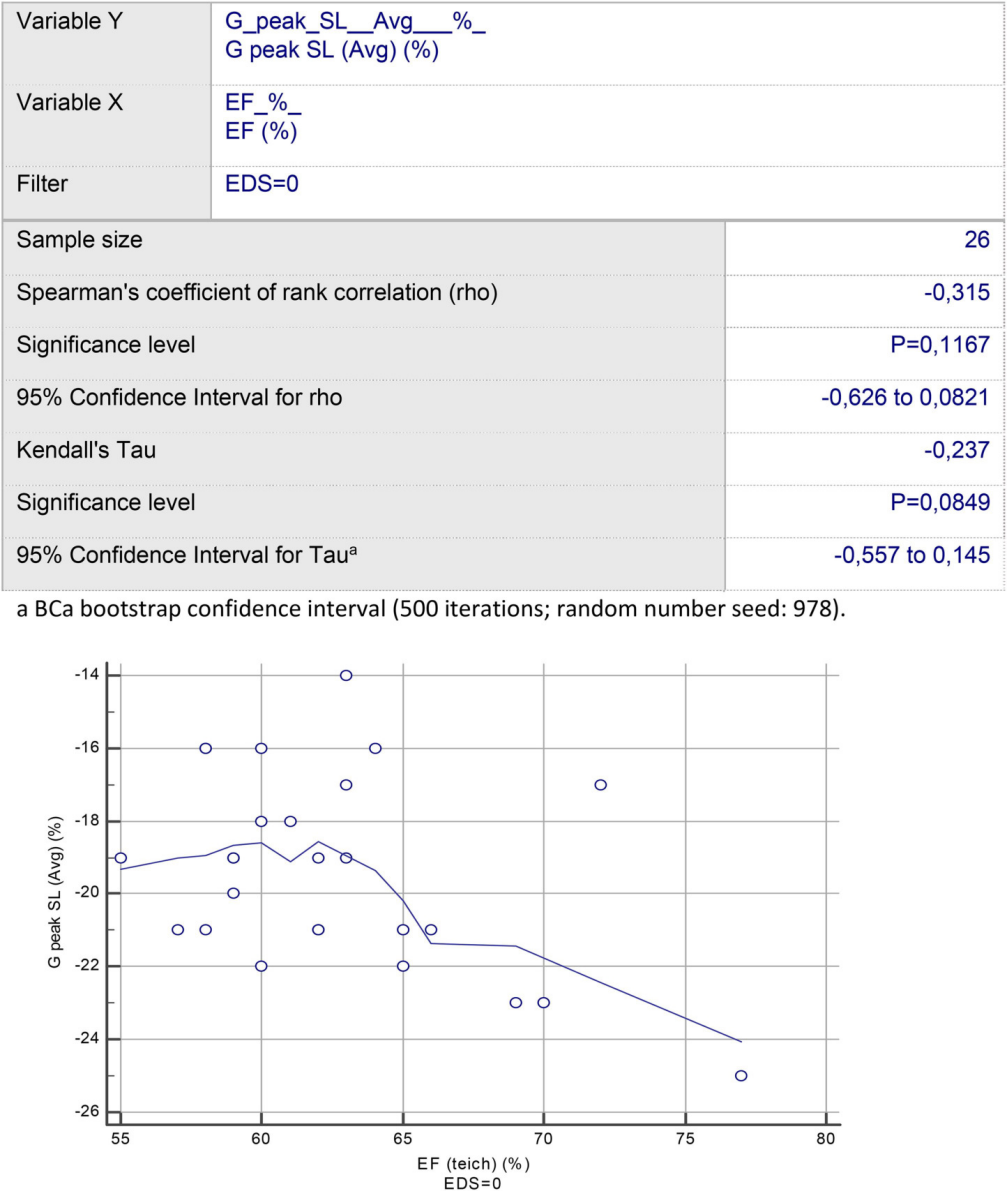
Rank correlation analysis reveals a statistically significant trend, suggesting that hEDS subjects with normal EF can have a reduced G peak LS (Avg).

**Figure 2:**
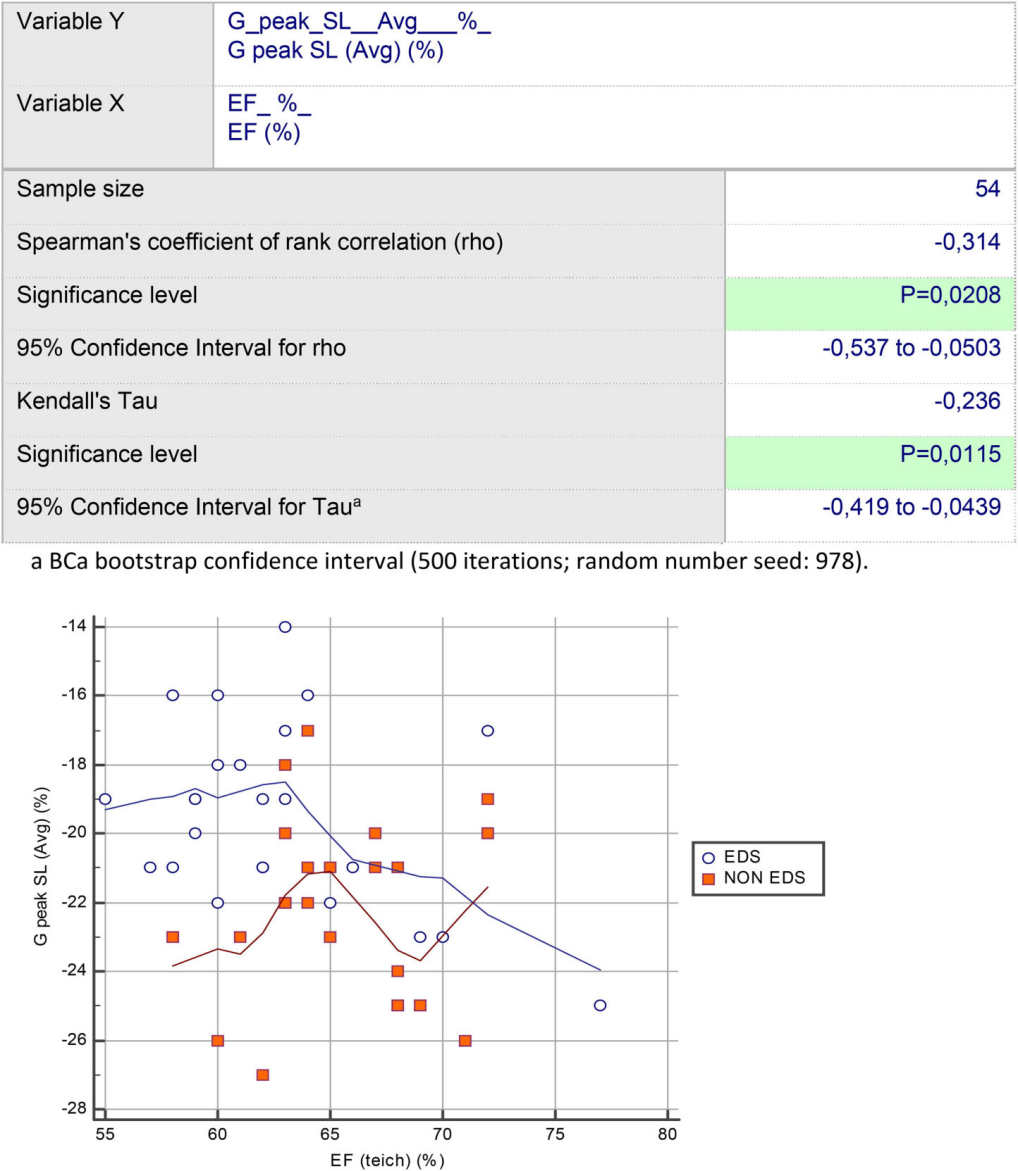
GLS (Avg) was statistically significantly correlated with EF among the entire cohort of subjects.

Rank correlation analysis revealed a statistically significant trend, suggesting that hEDS subjects with normal EF on conventional echocardiography can have a decrease in G peak LS Average during the strain study [Spearman's coefficient of rank correlation (rho): –0.315; significance level: *p* = 0.1167; 95% CI for rho: –0.626 to 0.0821]. This is different from the control group [Spearman's coefficient of rank correlation (rho): 0.065; significance level: *p* = 0.74; 95% CI for rho: –0.315 to 0.428] (Figure 1); indeed, G peak LS Avg was statistically significantly correlated with EF among the entire cohort of subjects [Spearman's coefficient of rank correlation (rho): –0.314; significance level: *p* = 0.0208; 95% CI for rho: –0.537 to –0.05] (Figure 2). Multivariable regression analysis demonstrated that the presence of hEDS was independently correlated with G peak LS (Avg) (*p* = 0.0004) (Table 5), but not with EF (*p* = 0.6673) (Table 6).

**Table 5:** Multiple regression of G peak Avg.

Independent variables	Coefficient	Std. Error	r_partial_	*t*	*P*
Regression Equation					
(Constant)	−15,0903				
BSA__m2_	5,0624	2,8706	0,2543	1,764	0,0846
EDS	−2,4119	0,9813	−0,3440	−2,458	0,0179
EF %_	−0,08734	0,08212	−0,1566	−1,064	0,2932
EDV__teich___ml_	−0,03797	0,03950	−0,1418	−0,961	0,3416
Ao Root__mm_	0,06755	0,1805	0,05569	0,374	0,7100
TAPSE__cm_	−0,1768	1,2612	−0,02090	−0,140	0,8891
Ascending_ Ao __mm_	−0,2836	0,1755	−0,2343	−1,616	0,1130
Ao Annulus__mm_	0,1349	0,2656	0,07550	0,508	0,6140

**Table 6:** Multiple regression of EF.

Independent variables	Coefficient	Std. Error	r_partial_	*t*	*P*
Regression Equation					
(Constant)	28,8552				
BSA__m2_	−1,2292	0,5323	−0,3322	−2,309	0,0258
EDS	0,1189	0,1902	0,09490	0,625	0,5352
FS__%_	1,0411	0,06515	0,9252	15,982	<0,0001
G_peak_SL__Avg___%_	0,008761	0,02596	0,05140	0,337	0,7374
EDV__teich___ml_	−0,1740	0,03947	−0,5578	−4,407	0,0001
SV__teich___ml_	0,2698	0,06374	0,5424	4,233	0,0001
TAPSE__cm_	0,3495	0,2259	0,2296	1,547	0,1292
Ascending Ao __mm_	0,04905	0,03087	0,2355	1,589	0,1193
Ao Annulus__mm_	−0,005407	0,04574	−0,01802	−0,118	0,9065
Ao Root__mm_	−0,03210	0,03098	−0,1561	−1,036	0,3060

## Discussion

Cardiovascular involvement in individuals diagnosed with hEDS—including MVP, impaired LV relaxation, and ARD—has been previously reported in the literature (10, 23). However, a comprehensive morphological and functional echocardiographic evaluation in pediatric patients has not yet been systematically performed. In the present study, TDI analysis revealed a reduction in late diastolic septal mitral velocity in the hEDS group, suggesting a mild impairment of LV diastolic function. This finding may reflect increased myocardial tissue stiffness in these patients. Moreover, a statistically significant difference in global ventricular function, as assessed by STE, was observed between hEDS patients and controls.

In normal myocardium, microfibrils are present both as free components within the connective tissue space and in association with collagen fibrils, elastic fibers, and basal lamina structures. They represent integral elements of the myocardial extracellular matrix and play a crucial role in anchoring extracellular matrix components to cardiomyocytes and capillaries (24). Microfibrils exert several essential functions, including providing a scaffold for tropoelastin deposition and elastic fiber assembly, and anchoring endothelial and epithelial cells to elastic fibers (25, 26). Although a statistically significant difference in LV ejection fraction (EF) between groups was observed, EF values remained within the normal range and are therefore unlikely to be clinically meaningful. In contrast, speckle-tracking echocardiography (STE) revealed a more pronounced statistical difference, with LV global longitudinal strain (GLS) values mildly reduced in pediatric patients with hEDS compared with controls. Importantly, these GLS values, while lower than in controls, remained within normal limits, suggesting that STE may detect subtle, subclinical myocardial functional changes that are not apparent in conventional EF assessments.

The correlation between LV global longitudinal strain (GLS) and ejection fraction (EF) in pediatric patients with hEDS was weak and may be influenced by individual outliers. This finding suggests that conventional EF can remain normal even when subtle reductions in myocardial deformation are present. Therefore, STE-derived GLS appears to be a more sensitive marker of early, subclinical myocardial involvement, capable of detecting changes that are not apparent with standard EF assessment.

The present study also demonstrates that MVP, traditionally described as a characteristic feature of adult hEDS (27, 28) and included in Criterion 2 of the 2017 International Classification of the Ehlers-Danlos Syndrome, is already detectable in childhood. Indeed, in this pediatric cohort, the prevalence of MVP was markedly higher than that reported in the general population (32% vs. 2%–5%) (29). This finding may be related to alterations in valvular collagen composition, leading to an imbalance between the tensile forces exerted by the chordae tendineae and the papillary muscles, along with the increased elastic resistance of the valve leaflets. Owing to their extensibility, microfibrils contribute to the mechanical properties of mature elastic tissues by redistributing mechanical load among individual elastic fibers (25, 26). These structures are localized at the tips of the papillary muscles, where they originate from the ends of the myocytes, and are believed to transmit contractile force from the myocytes to the collagen fibers of the chordae tendineae (24).

An increase in AR diameters has been previously described in adult patients with hEDS (18). Although the pediatric cohort evaluated in the present study exhibited mean *Z*-scores of the SV and AA within physiological limits, seven patients (25%) demonstrated AA dilation—five of a mild degree (*Z*-score 2.01–3.0) and one of a moderate degree (*Z*-score 3.01–4.0). These findings suggest that aortic involvement may already be present in childhood. Notably, despite *Z*-score values of the AR and AA remaining within the normal range, statistically significant differences in these parameters were observed between hEDS patients and controls (*p* = 0.016 and *p* = 0.0016, respectively) (Table 2).

According to existing evidence, type III collagen plays a pivotal role in fibrillogenesis and, secondarily, in the development of the cardiovascular system, intestine, skin, and joints. Deficiency of type III collagen leads to the abnormal development and impaired function of these organs (30). Under physiological conditions, type III collagen is not only a key structural component of fibrils in tissues such as the aortic tunica media but also acts as a regulatory factor in type I collagen fibrillogenesis. Specifically, type III collagen modulates the diameter of type I collagen fibrils in a tissue- and development-dependent manner (31).

The observed findings may therefore be explained by alterations in type III collagen interactors and, subsequently, by disrupted interactions between collagen and microfibrils through the regulation of type I collagen. These alterations may lead to myocardial structural modifications that account for the slightly reduced myocardial deformation parameters observed in our cohort, suggesting the presence of early subclinical LV dysfunction.

Recent studies on cell trafficking emphasize its role in extracellular matrix organization and tissue development. In hEDS, disruptions in protein transport and matrix assembly may contribute to early cardiovascular alterations, including mitral valve prolapse and subtle myocardial dysfunction, linking molecular defects to the clinical findings observed in our pediatric cohort (32).

This study has some limitations, primarily due to the relatively small sample size of the cohort. Due to the rarity of pediatric hEDS, the number of patients available for inclusion was limited, and a formal power analysis could not be performed. This represents a study limitation, and the results should be interpreted with caution, although they provide valuable preliminary insights into early cardiovascular alterations in this population. Consequently, the observed morphological and functional cardiac findings should be validated in larger populations. From a diagnostic perspective, although speckle-tracking echocardiography provides sensitive detection of subclinical myocardial dysfunction, strain measurements may be influenced by image quality, vendor-specific software variability, and the lack of universally established pediatric reference values. In addition, the retrospective nature of the study limits our ability to assess disease progression. Prospective studies with longitudinal echocardiographic follow-up would allow for the evaluation of the temporal evolution of cardiovascular manifestations, such as ARD and impaired myocardial function.

In conclusion, the present study demonstrates that cardiovascular abnormalities traditionally described in adults with hypermobile Ehlers-Danlos syndrome (hEDS), including MVP and mild aortic dilatation, can be detected in children. Although overt structural abnormalities remain relatively uncommon and global systolic function, as assessed by EF, is preserved, advanced echocardiographic techniques—particularly STE—identified subtle reductions in myocardial deformation parameters, suggesting early subclinical left ventricular dysfunction. These findings support the hypothesis that extracellular matrix alterations may affect myocardial and valvular structure from early life. Therefore, a comprehensive echocardiographic evaluation, including strain analysis, may represent a valuable, non-invasive strategy for early cardiovascular surveillance in pediatric patients with hEDS. Larger prospective longitudinal studies are warranted to clarify the clinical significance and long-term evolution of these early alterations and to optimize risk stratification and follow-up protocols.

## Data Availability

The raw data supporting the conclusions of this article will be made available by the authors, without undue reservation.
